# Contributions of the international plant science community to the fight against human infectious diseases – part 1: epidemic and pandemic diseases

**DOI:** 10.1111/pbi.13657

**Published:** 2021-07-19

**Authors:** Maria Lobato Gómez, Xin Huang, Derry Alvarez, Wenshu He, Can Baysal, Changfu Zhu, Victoria Armario‐Najera, Amaya Blanco Perera, Pedro Cerda Bennasser, Andera Saba‐Mayoral, Guillermo Sobrino‐Mengual, Ashwin Vargheese, Rita Abranches, Isabel Alexandra Abreu, Shanmugaraj Balamurugan, Ralph Bock, Johannes.F. Buyel, Nicolau B. da Cunha, Henry Daniell, Roland Faller, André Folgado, Iyappan Gowtham, Suvi T. Häkkinen, Shashi Kumar, Sathish Kumar Ramalingam, Cristiano Lacorte, George P. Lomonossoff, Ines M. Luís, Julian K.‐C. Ma, Karen. A. McDonald, Andre Murad, Somen Nandi, Barry O’Keefe, Kirsi‐Marja Oksman‐Caldentey, Subramanian Parthiban, Mathew J. Paul, Daniel Ponndorf, Elibio Rech, Julio C. M. Rodrigues, Stephanie Ruf, Stefan Schillberg, Jennifer Schwestka, Priya S. Shah, Rahul Singh, Eva Stoger, Richard M. Twyman, Inchakalody P. Varghese, Giovanni R. Vianna, Gina Webster, Ruud H. P. Wilbers, Teresa Capell, Paul Christou

**Affiliations:** ^1^ Department of Crop and Forest Sciences University of Lleida‐Agrotecnio CERCA Center Lleida Spain; ^2^ Instituto de Tecnologia Química e Biológica António Xavier Universidade Nova de Lisboa Oeiras Portugal; ^3^ Plant Genetic Engineering Laboratory Department of Biotechnology Bharathiar University Coimbatore India; ^4^ Max Planck Institute of Molecular Plant Physiology Potsdam‐Golm Germany; ^5^ Fraunhofer Institute for Molecular Biology and Applied Ecology IME Aachen Germany; ^6^ Institute for Molecular Biotechnology RWTH Aachen University Aachen Germany; ^7^ Centro de Análise Proteômicas e Bioquímicas de Brasília Universidade Católica de Brasília Brasília Brazil; ^8^ School of Dental Medicine University of Pennsylvania Philadelphia PA USA; ^9^ Department of Chemical Engineering University of California, Davis Davis CA USA; ^10^ Industrial Biotechnology and Food Solutions VTT Technical Research Centre of Finland Ltd Espoo Finland; ^11^ International Centre for Genetic Engineering and Biotechnology New Delhi India; ^12^ Brazilian Agriculture Research Corporation Embrapa Genetic Resources and Biotechnology and National Institute of Science and Technology Synthetic in Biology Parque Estação Biológica Brasilia Brazil; ^13^ Department of Biological Chemistry John Innes Centre Norwich UK; ^14^ Institute for Infection and Immunity St. George’s University of London London UK; ^15^ Global HealthShare Initiative University of California, Davis Davis CA USA; ^16^ Molecular Targets Program Center for Cancer Research, National Cancer Institute, and Natural Products Branch Developmental Therapeutics Program Division of Cancer Treatment and Diagnosis National Cancer Institute, NIH Frederick MD USA; ^17^ Institute for Phytopathology Justus‐Liebig‐University Giessen Giessen Germany; ^18^ Institute of Plant Biotechnology and Cell Biology University of Natural Resources and Life Sciences Vienna Austria; ^19^ Department of Microbiology and Molecular Genetics University of California, Davis Davis CA USA; ^20^ TRM Ltd Scarborough UK; ^21^ Laboratory of Nematology Plant Sciences Group Wageningen University and Research Wageningen The Netherlands; ^22^ ICREA Catalan Institute for Research and Advanced Studies Barcelona Spain

**Keywords:** Molecular farming, plant‐made pharmaceuticals, HIV/AIDS, SARS‐CoV‐2, COVID‐19

## Abstract

Infectious diseases, also known as transmissible or communicable diseases, are caused by pathogens or parasites that spread in communities by direct contact with infected individuals or contaminated materials, through droplets and aerosols, or via vectors such as insects. Such diseases cause ˜17% of all human deaths and their management and control places an immense burden on healthcare systems worldwide. Traditional approaches for the prevention and control of infectious diseases include vaccination programmes, hygiene measures and drugs that suppress the pathogen, treat the disease symptoms or attenuate aggressive reactions of the host immune system. The provision of vaccines and biologic drugs such as antibodies is hampered by the high cost and limited scalability of traditional manufacturing platforms based on microbial and animal cells, particularly in developing countries where infectious diseases are prevalent and poorly controlled. Molecular farming, which uses plants for protein expression, is a promising strategy to address the drawbacks of current manufacturing platforms. In this review article, we consider the potential of molecular farming to address healthcare demands for the most prevalent and important epidemic and pandemic diseases, focussing on recent outbreaks of high‐mortality coronavirus infections and diseases that disproportionately affect the developing world.

## Introduction

In the first years of the 21st century, infectious diseases are still responsible for ˜17% of all human deaths (Vos *et al*., 2020). The impact of infectious diseases as a proportion of all deaths has been falling decade on decade thanks to improvements in hygiene, vaccination and health care, but infectious diseases are still a major burden on national health systems. An infectious disease is defined as a transmissible disease caused by pathogens (viruses, bacteria or fungi) or parasites (both microbial and invertebrate, primarily endoparasites) but usually excludes infestations with arthropods such as ticks and lice, which typically act as ectoparasites.

An **epidemic disease** is an infectious disease that may or may not be endemic in a population but spreads rapidly among communities of certain geographic areas (CDC, 2020b). Epidemics can be contained using a combination of natural and technological factors (including therapy and vaccination) that prevent spreading to other regions. Currently, there are many epidemic diseases: cholera in Yemen (WHO, 2019), measles in Burundi (WHO, 2020g), measles and Ebola in the Democratic Republic of the Congo (WHO, 2020b,c), Ebola in Uganda (WHO, 2020e), measles in the Philippines, Kuala Koh, Samoa and New Zealand (Ministry of Health NZ, 2020) and dengue in parts of Southeast Asia and South America (WHO, 2017a). The difference between an outbreak and an epidemic is often one of scale, but each is typified by a rapid increase in cases above the typical baseline incidence. The major difference between an endemic and epidemic disease is the unexpected nature of the epidemic, and each therefore requires different tactical and strategic approaches for control. A **pandemic disease** is an epidemic that spreads over multiple large regions or worldwide (CDC, 2020b), and such diseases are more common today than in previous centuries because of the pervasive nature of global travel and trade (Morens *et al*., 2009). Although the term pandemic is often associated with rapidly spreading diseases such as influenza and the current COVID‐19 pandemic caused by severe acute respiratory syndrome‐associated coronavirus 2 (SARS‐CoV‐2), which are transmitted by droplets or aerosols (CDC, 2020a; Morawska and Milton, 2020; WHO, 2020a), it also applies to diseases such as HIV/AIDS caused by human immunodeficiency virus, which is transmitted more slowly via body fluids (WHO, 2020f).

This review article provides a critical assessment of the use of plant biotechnology as a means to tackle epidemic and pandemic diseases, focussing on the use of plants as bioreactors for the production of research reagents and pharmaceutical products including vaccines, antibodies and antivirals. The diseases covered by the article are summarized in Table 1, with more details on the incidence, prevalence and burden associated with these diseases provided in Table S1.

**Table 1:** Classification of epidemic and pandemic diseases based on their epidemiology, showing the number of people affected in a specific time and place. The fatality rate was calculated by dividing the number of deaths by the total number of identified cases in the specific time and place. References are listed in Table S1. Viral strains are underlined

Classification	Disease	Number of people affected	Fatality rate
Epidemic	Ebola	34 559 (Africa, 1976 – June 2020)	44.1%
	Zika	200 000 (the Americas, 2016)	Brazil, 2016: 8.3%
Pandemic	SARS	8098 (worldwide, November 2002 – July 2003)	9.6%
	MERS	2516 (worldwide, April 2012 – January 2020)	34.3%
	COVID‐19	More than 180 000 000 (worldwide, January 2020 – July 2021)	2.1%
	H5N1 and H7N9 influenza	H5N1: 862 (worldwide, 2003–2020) H7N9: 1565 (worldwide, 2017 – August 2020)	H5N1: 53% H7N9: 39%
	Hepatitis	HAV: 1.4 million/year (worldwide estimate) HBV: 257 million (worldwide estimate, 2020) HCV: 71 million (worldwide, 2015) HDV: 5% of patients with HBV (worldwide estimate) HEV: 20 million/year (worldwide estimate)	HAV: 0.3–0.6% HBV: 0.35% HCV: 0.56% HBV + HDV: 1% HEV: 0.22% (2015)
	HIV/AIDS	38 million (worldwide, up to 2019)	1.8%
	HPV	528 000 (worldwide estimate, 2012)	20.4%
	Seasonal influenza	450 000 (worldwide, January–May 2020)	0.11%

COVID‐19, coronavirus disease 2019; H5N1/H7N9, influenza strains (hemagglutinin/neuraminidase); HAV/HBV/HCV/HDV/HEV, hepatitis A/B/C/D/E virus; HIV/AIDS, human immunodeficiency virus/acquired immunodeficiency syndrome; HPV, human papillomavirus; MERS, Middle East respiratory syndrome; SARS, severe acute respiratory syndrome.

## Why plants?

Plant biotechnology has much to offer in the fight against infectious diseases, from the provision of emergency testing infrastructure (Webb *et al*., 2020) to the manufacture of small‐molecule drugs, recombinant antivirals, subunit vaccines, engineered viruses and virus‐like particle (VLP) vaccines, therapeutic proteins, antibodies and diagnostic reagents (Capell *et al*., 2020; Daniell *et al*., 2016, 2019, 2021; McDonald and Holtz, 2020; Rosales‐Mendoza, 2020; Tusé *et al*., 2020). Although plants have been used as a platform for the production of pharmaceutical proteins for more than 30 years (Fischer and Buyel, 2020; Ma *et al*., 2003a), their potential advantages in terms of scale and speed were highlighted by the slow response of traditional manufacturing platforms to epidemics of severe acute respiratory syndrome (SARS) in 2002/2003, H1N1/09 influenza in 2009, Middle East respiratory syndrome (MERS) in 2012, Ebola in 2014/2015 and Zika in 2016/2017 (Bradley and Bryan, 2019; Kobres *et al*., 2019). Plants are still more often described as ‘promising’ or ‘**emerging**’ platforms rather than genuine alternatives, but their potential for the large‐scale production of reagents and vaccines has been demonstrated in the context of COVID‐19, particularly through the clinical testing of VLP‐based vaccines (Ward *et al*., 2021a).

The ability of plants to produce pharmaceutical proteins has been demonstrated in hundreds of proof‐of‐principle studies and in a growing number of clinical trials, with a small number of products reaching the market as approved biologics or medical devices (Fischer and Buyel, 2020; Ma *et al*., 2003a; Ward *et al*., 2020). Additional proteins have been expressed as diagnostics or research reagents, due to the shorter development times and lower regulatory burden (Tschofen *et al*., 2016). This niche discipline, known as molecular farming, was initially promoted by citing three key advantages of plants over fermenter‐based alternatives such as bacteria, yeast and mammalian cells: low cost, greater scalability and intrinsic safety (Mir‐Artigues *et al*., 2019; Nandi *et al*., 2016). The cultivation of plants in greenhouses (or even the field, where permitted) is much less expensive than fermenters and can be realized on a much larger scale. Plants are also intrinsically unable to support the replication of human viruses, effectively behaving as self‐contained disposable bioreactors. However, the early development of plant‐based pharmaceuticals was limited by four main drawbacks that reduced industrial confidence: low yields, high purification costs, regulatory barriers and plant‐specific glycans. The second wave of molecular farming addressed these issues by increasing yields, optimizing downstream processing (Buyel *et al*., 2015; Peyret *et al*., 2019; Zischewski *et al*., 2015) for enhanced recovery and purification (Box 1), engaging with regulators to develop new guidelines (Ma *et al*., 2015) and devising strategies to remove or modify plant glycans where this improved product functions, while also showing in multiple clinical trials that they pose no significant risk (Schroberer and Strasser, 2018). Plant‐based production systems now have the potential to compete with microbial and mammalian cells in fermenters, including the integration of pharmaceutical good manufacturing practice (GMP) at least for the downstream processing (DSP) steps (Fischer *et al*., 2012; Ma *et al*., 2015). Furthermore, the ease of transporting seeds and expression constructs, combined with the inexpensive cultivation and increasing availability of disposable equipment and portable infrastructure, means that plants can be grown where needed to provide a local source of research reagents, and potentially also vaccines or drugs, even in developing country settings.

Box 1 **Recovery and purification of biopharmaceutical proteins from plants and plant cell cultures**

The large‐scale manufacturing of biopharmaceuticals is often more readily achievable using plants rather than fermenter‐based systems, but this places additional pressure on the downstream processing (DSP) steps (Buyel *et al*., 2017). The latter stages of DSP are more dependent on the product than the production system; hence, the same platform chromatography steps can be applied to antibodies produced in mammalian cells and plants (Ma *et al*., 2015). However, the early stages of DSP must deal with challenges specific to the recovery and purification of biopharmaceuticals from plant tissues, because most molecular farming products are retained within the plant cell or in the apoplast, with relatively few products secreted fully into the medium of cell suspension/hairy root cultures or into the hydroponic fluid in rhizosecretion platforms (Drake *et al*., 2009; Madeira *et al*., 2016a,b). Cost‐efficient conditioning and pre‐processing steps have therefore been developed to address plant‐specific challenges (product recovery from leaf or seed tissue, which results in a much higher burden of particles, fibres and host cell proteins than other systems), including precipitation, flocculation, optimized filter trains and membrane‐based purification (Buyel and Fischer, 2014; Hassan *et al*., 2008; Menzel *et al*., 2016; Opdensteinen *et al*., 2019). The DSP steps for whole plants and plant cell cultures have evolved to a similar process scale and level of professionalism as established for conventional host systems (Schillberg *et al*., 2019). A framework has also been established to address process design and development, consisting of models for process steps (Buyel, 2016), overall layout (Nandi *et al*., 2016) and costs (Buyel and Fischer, 2012; McNulty *et al*., 2020; Walwyn *et al*., 2015). Accordingly, manufacturing vaccines and therapeutics in plant‐based systems is now a scalable and economically viable alternative to traditional production systems (Tusé *et al*., 2020).

**Figure 1:**
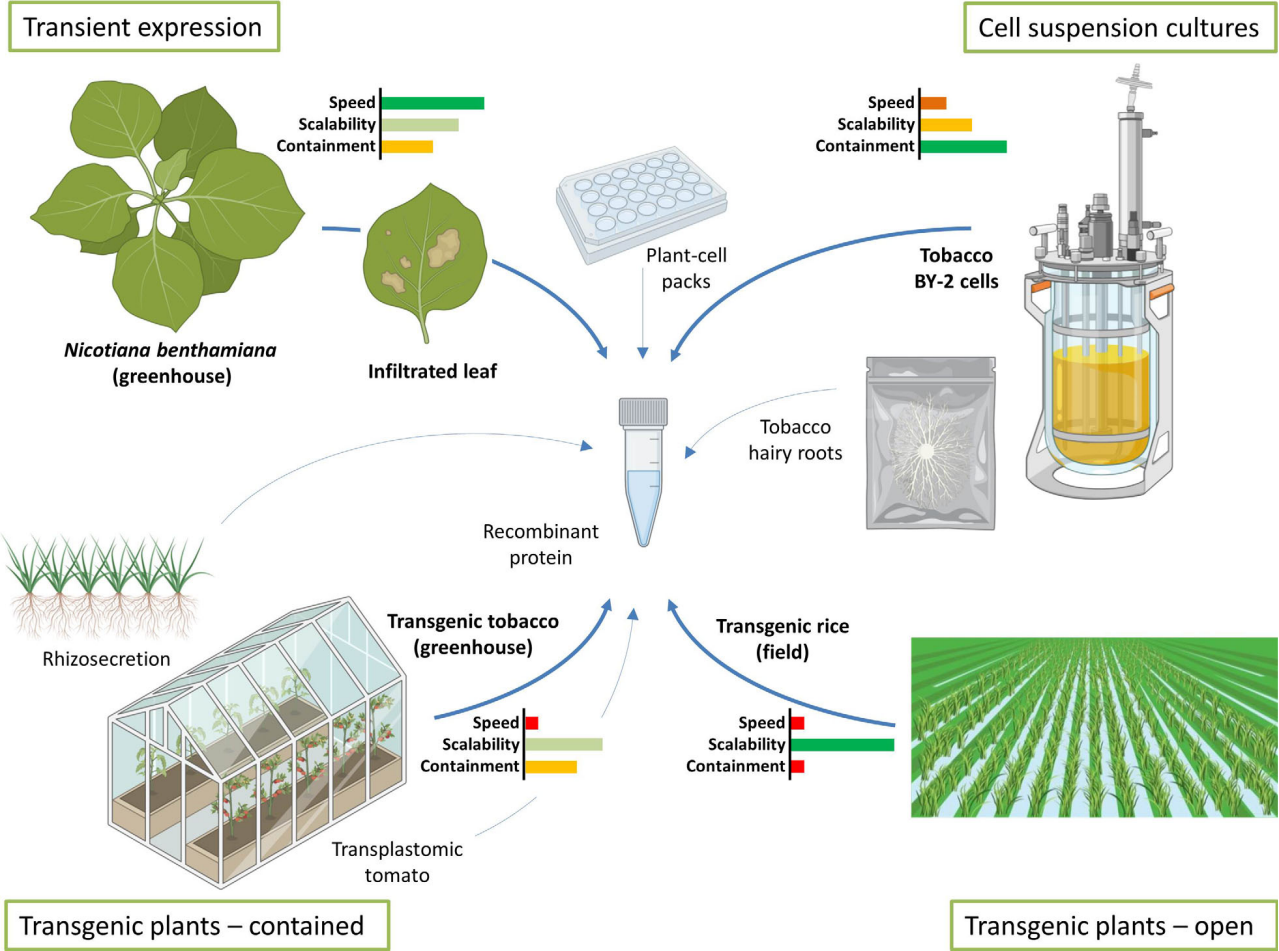
The three major molecular farming platforms are transient expression, transgenic plant cell suspension cultures and transgenic plants (Huebbers and Buyel, 2021), the latter either grown in containment or in the open field (bold text, thick arrows). The relative advantages and disadvantages of the three platforms are shown in terms of speed (the faster the better), scalability (the larger the better, generally inversely related to costs) and containment (the more contained the lesser the regulatory burden) with separate indicators for transgenic plants grown indoors and outdoors. Four additional minor or **emerging** platforms are also shown (regular text, thin arrows). Plant cell suspension cultures are usually transgenic cell lines, but transient expression is also possible (Sukenik *et al*., 2018) and has been realized in the form of plant cell packs for the high‐throughput and highly automated testing of expression constructs with immediately scalable expression (Gengenbach *et al*., 2020; Rademacher *et al*., 2019). Transgenic organ cultures such as hairy roots can be regarded as an extension of the cell suspension culture concept because the organ cultures are likewise grown in containment in bioreactors (Doran, 2013; Wongsamuth and Doran, 1997). Variants on the theme of transgenic plants include transplastomic plants, where the transgene is inserted into the plastid genome rather than the nuclear genome (Bains *et al*., 2017; Berecz *et al*., 2017; Bock, 2015; Zhang *et al*., 2017), and rhizosecretion, in which proteins are secreted by the roots of plants into the hydroponic medium, so that aggressive extraction methods are unnecessary (Drake *et al*., 2009; Madeira *et al*., 2016a,b). The figure includes images from Biorender (https://biorender.com/).

The renaissance of molecular farming has led to three major platforms (Figure 1): transient expression (mostly in *Nicotiana benthamiana*), transgenic plants (mostly tobacco and cereals, but also fruit and vegetable crops, legumes and oilseeds) and plant cell suspension cultures (mostly tobacco and rice), which can be extended to include other clonally propagated platforms in containment such as algae, moss, duckweed and plant organ cultures (e.g. hairy roots), which are thus covered by the same regulatory guidelines as cell cultures. These platforms have all been used to produce pharmaceutical proteins targeting infectious diseases because they have advantages for different disease types, which we explore in more detail when we consider the diseases in turn. Briefly, however, transient expression involves the infiltration of wild‐type plants or plant cells with bacteria or their infection with viral vectors, leading to a burst of recombinant protein production. This allows protein recovery after a few days, and the entire platform is therefore extremely rapid and scalable. This is ideal for rapid responses in the face of **emerging** epidemic and pandemic diseases, and several companies have already invested in large‐scale production facilities using *N. benthamiana* to develop vaccines against seasonal and pandemic influenza (Pillet *et al*., 2016, 2019; Shoji *et al*., 2011; Ward *et al*., 2020), Ebola (Hiatt *et al*., 2015), and most recently COVID‐19 (Capell *et al*., 2020; Ward *et al*., 2021a). The scalability and response time were succinctly demonstrated in a DARPA Blue Angel program that produced 10 million doses of influenza vaccine in one month (Lomonossoff and D’Aoust, 2016). In the fight against COVID‐19, transient expression has been successful for the development of diagnostic reagents and the components of assay kits (Capell *et al*., 2020) and also for the development of vaccines, with the Canadian government already placing orders for 76 million doses of the Medicago VLP vaccine (Ward *et al*., 2021a). It takes longer to develop products when the proteins are expressed in transgenic plants, but this allows the continuous, large‐scale production of proteins, which is ideal for slowly spreading or established pandemic diseases, as well as widely prevalent **endemic diseases** discussed in our sister article (He *et al*., 2021), and where there is a large demand for biologics and insufficient capacity in the current supply chain. Tobacco is widely used to develop transgenic lines expressing pharmaceutical proteins, but leafy crops have the disadvantage of product instability during storage, meaning that immediate extraction and downstream purification is necessary unless the biomass can be frozen or dried (Hoelscher *et al*., 2018). Freeze‐dried leaves can be stored at ambient temperature for up to 3–4 years (Daniell *et al*., 2020; Herzog *et al*., 2017; Park *et al*., 2020; Su *et al*., 2015), and seeds have been stored at ambient temperatures even longer without loss of protein activity, so both systems obviate the need for an expensive cold chain, which is especially valuable in remote areas in developing countries (Sabalza *et al*., 2013; Stoger *et al*., 2005). Furthermore, the use of any edible tissue for recombinant protein expression provides the option for oral delivery with zero (Daniell *et al*., 2020; Herzog *et al*., 2017; Park *et al*., 2020; Su *et al*., 2015; Tacket *et al*., 1998, 2000) or minimal processing (Nandi *et al*., 2005; Zavaleta *et al*., 2007) or topical application as a crude extract (Ramessar *et al*., 2008a) which eliminates up to 80% of the costs of production, as discussed in greater detail in our sister article (He *et al*., 2021). The ability to use crude extracts also allows further cost savings by producing multiple proteins simultaneously within the same plants, as recently shown for two lectins and an HIV‐specific antibody as the basis for a microbicidal cocktail to prevent HIV transmission (Vamvaka *et al*., 2018). Finally, clonal systems such as cell suspension cultures are closest to traditional cell‐based platforms in terms of GMP manufacturing and were therefore pioneers in the clinical development of plant‐made pharmaceuticals. The first approved pharmaceutical product (taliglucerase alfa, a recombinant version of glucocerebrosidase indicated for Gaucher’s disease) was manufactured in carrot cell suspension cultures (Tekoah *et al*., 2015). Plant cells suffer similar limitations to other fermenter systems in terms of scalability (Santos *et al*., 2016). Therefore, plant cells and similar systems with lower scalability are more suited to the production of pharmaceuticals for orphan diseases and others with relatively low prevalence, such as specific forms of cancer, although recent studies have shown how scalability can be increased by semi‐continuous production (Macharoen *et al*., 2021). Like transgenic plant tissues, plant cell pellets can be administered orally or crude extracts can be applied topically to reduce processing costs, so they may yet find use for the production of oral vaccines and other non‐injected products. The cost implications of the different molecular farming platforms have been addressed in multiple techno‐economic assessments (Alam *et al*., 2018; Corbin *et al*., 2020; McNulty *et al*., 2020; Mir‐Artigues *et al*., 2019; Nandi *et al*., 2016; Schillberg *et al*., 2019).

## Small‐molecule drugs

**Figure 2:**
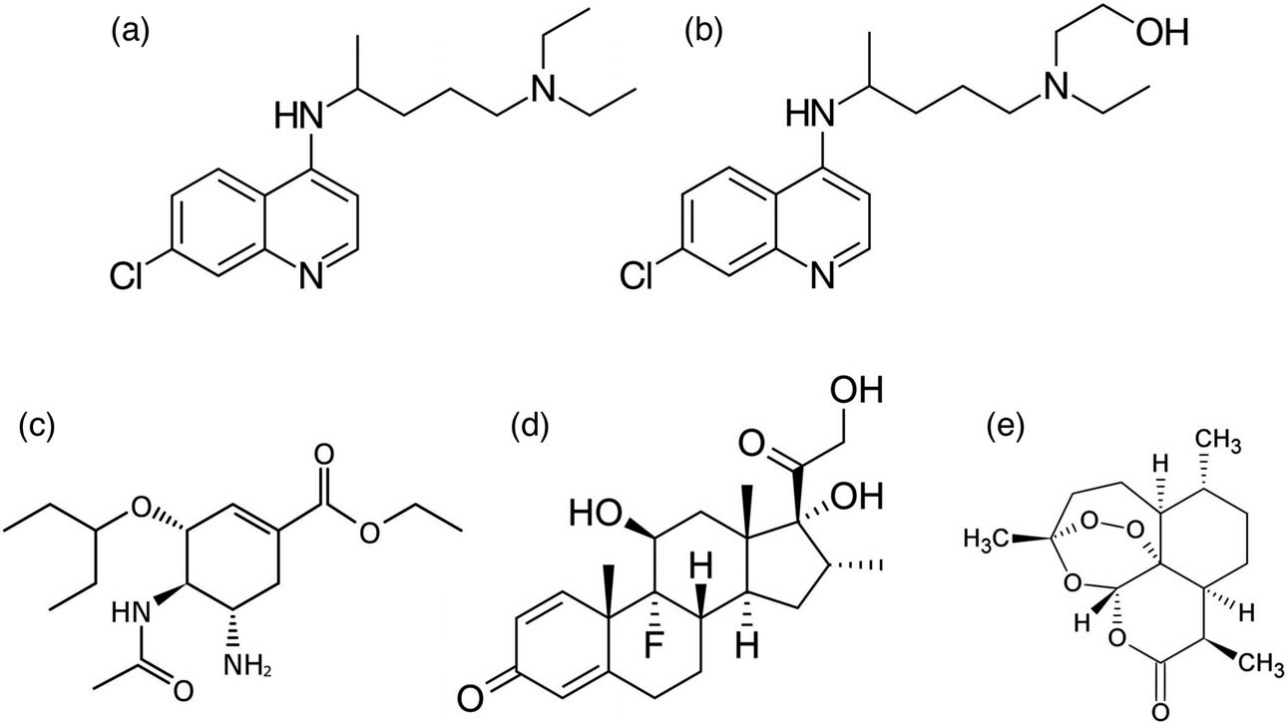
Chemical structures of (a) chloroquine, (b) hydroxychloroquine, (c) oseltamivir, (d) dexamethasone and (e) artemisinin

In addition to the production of pharmaceutical proteins, plants can also be used to manufacture small‐molecule drugs, including antivirals. Interest in antivirals derived from wild‐type plants has increased following the discovery that medicinal plant extracts containing polyphenols, terpenes, cumarins and alkaloids often show antiviral properties (Daglia, 2012; Denaro *et al*., 2020). Such molecules can inhibit viral entry, endosome and lysosome acidification (required for uncoating) or replication (Ben‐Shabat *et al*., 2020; Musarra‐Pizzo *et al*., 2019; Shin *et al*., 2010). More than one third of the 185 antiviral drugs approved in the last 38 years are natural products or their derivatives (Newman and Cragg, 2020). Indeed, COVID‐19 has renewed interest in antiviral natural products, including heterocyclic molecules such as chloroquine, hydroxychloroquine, oseltamivir and dexamethasone (Figure 2). Chloroquine (originally isolated from the bark of the cinchona tree, now produced synthetically) and its derivative hydroxychloroquine are antimalarial drugs that also inhibit the glycosylation of viral proteins (Savarino *et al*., 2004). However, the efficacy and safety of these drugs for the treatment of COVID‐19, though widely reported, has not been confirmed (Cortegiani *et al*., 2020). Similarly, artemisinic acid derivatives (also widely used for the treatment of malaria, see our sister article in this issue, He *et al*., 2021) can be produced in plants as artemisinic acid (Fuentes *et al*., 2016, 2018) or artemisinin (Malhotra *et al*., 2016) and appear to offer potential for the treatment of COVID‐19 (Gendrot *et al*., 2020). Oseltamivir is a neuraminidase inhibitor derived from shikimic acid (originally extracted from plants, now produced in bacteria) that has been used to treat multiple viral diseases including most recently COVID‐19 (Chhikara *et al*., 2020). Dexamethasone, which is recommended for critically ill COVID‐19 patients in a hospital setting (Liffey, 2020), is also produced by chemical synthesis, but the corticosteroid backbone is found in many plant‐derived steroids and could be used as an alternative source for larger‐scale production (Patel and Savjani, 2015).

Plant‐derived small molecules have also been screened to find new products that inhibit the SARS‐CoV‐2 main protease, revealing the antiviral activity of luteolin‐7‐glucoside, demethoxycurcumin, apigenin‐7‐glucoside, oleuropein, curcumin, catechin and epicatechin gallate (Khaerunnisa *et al*., 2020). Similarly, screening a library of 100 FDA‐approved antiviral compounds and 1000 active components from Indian medicinal plants showed that anthraquinone rhein, ergosterol withanolide D, sterol lactone withaferin A, fluoroquinolone enoxacin and aloe‐emodin can also bind to the protease (Kumar *et al*., 2020).

## Molecular farming as a strategy to address rapidly spreading epidemic and pandemic diseases

### Coronavirus respiratory diseases

Coronaviruses are encapsulated single‐stranded positive‐sense RNA viruses that typically cause mild respiratory diseases such as the common cold. However, several novel strains of β coronavirus have emerged over the last 20 years that can trigger fatal acute respiratory infections, particularly in people with pre‐existing health conditions. Major outbreaks occurred in 2002/2003 (severe acute respiratory syndrome‐associated coronavirus, SARS‐CoV), 2012 (Middle East respiratory syndrome‐associated coronavirus, MERS‐CoV) and most recently in 2019 (SARS‐CoV‐2) (Bradley and Bryan, 2019; Kobres *et al*., 2019; Park, 2020). The latter was classified as a pandemic by the WHO on 11 March 2020 (WHO Director‐General, 2020) and has thus far infected more than 180 million people worldwide causing ˜3.9 million deaths (Johns Hopkins University of Medicine, 2021) although the recent approval of several vaccines is now providing hope that the disease can at last be brought under control (Hooker and Palumbo, 2020). The three most lethal β coronaviruses and the properties of the associated diseases are compared in Table 2.

**Table 2:** Properties of the three most lethal β coronavirus infections (modified from Park, 2020). R_0_ is the basic reproduction number, the expected number of cases directly generated by one case in a population where all individuals are susceptible to the disease

	SARS‐CoV	MERS‐CoV	SARS‐CoV‐2 (COVID‐19)
Period	2002–2003	2012– (ongoing)	2019– (ongoing)
Natural host	Bats	Bats	Bats
Intermediate host	Civet cats among others	Dromedary camels	Unknown
Transmission method	Respiratory droplets, direct contact	Respiratory droplets, direct contact	Respiratory droplets, direct contact
R_0_	2*–*5	<1	2*–*3
Incubation period	4.6 days	5.2 days	5.1 days
Case fatality rate	9.6%	34.4%	2.1%
Most common symptoms	High fever (>38°C) headache, an overall feeling of discomfort, and body aches	Fever, chills, generalized myalgia, cough, shortness of breath, nausea, vomiting and diarrhoea	Cough, shortness of breath or difficulty breathing, fever, chills, muscle pain, sore throat, new loss of taste or smell

Molecular farming played a negligible role in the fight against SARS and MERS because these epidemics, while serious, did not reach the pandemic status of COVID‐19, and the technology was in any case not ready for a large‐scale response. Nevertheless, the N‐terminal ectodomain of the SARS‐CoV spike (S) protein was expressed in tobacco, lettuce and tomato and was in some cases proven to be immunogenic (Li and Chye, 2009; Li *et al*., 2006; Pogrebnyak et al., 2005). Furthermore, the SARS‐CoV membrane (M) protein and nucleocapsid (N) protein were produced by transient expression in *N. benthamiana*, again providing evidence of immunogenicity (Demurtas *et al*., 2016; Zheng *et al*., 2009). The yield of the N protein was 79 mg/kg fresh leaf biomass (Zheng *et al*., 2009).

The outbreak of COVID‐19 resulted in a much more significant reaction from the molecular farming community, in part reflecting the rapid spread of the disease and the need for urgent responses, and in part reflecting the profound growth of plant‐based transient expression as a large‐scale vaccine manufacturing platform in the 18 years since SARS (Capell *et al*., 2020; Rosales‐Mendoza, 2020). From the outset, molecular farming was acknowledged as a potential response platform and attracted a substantial amount of investment. This was true especially for the production of research‐grade reagents and vaccine candidates, but also for the development of therapeutics, including antibodies against the virus and the hyperactive immune response linked to the most severe and often fatal symptoms of COVID‐19. Because research‐grade reagents do not need regulatory clearance or clinical testing, many current academic and commercial researchers in the molecular farming community (including the authors of this article) stepped up following the outbreak of COVID‐19 in order to use their technology to produce SARS‐CoV‐2 proteins, antibodies for their detection and PCR control reagents. These products can also double as clinical‐grade products (vaccine candidates and therapeutics) if produced under GMP conditions.

Perhaps the most important target of molecular farming in the context of COVID‐19 is the SARS‐CoV‐2 trimeric S protein and derivatives such as the 223‐residue S_B_ receptor‐binding domain (RBD). The full S protein is 1255 amino acids in length (˜180 kDa), which is cleaved into the receptor‐binding S1 subunit (˜110 kDa) and the fusion‐promoting S2 subunit (˜70 kDa). The S protein contains 22 sites for *N*‐linked glycans (19 of which have been experimentally confirmed), 13 in the S1 subunit and low levels of *O*‐linked glycosylation (Ramírez Hernández *et al*., 2021), which increase its molecular weight. The RBD has a molecular weight of ˜35 kDa. These proteins can be developed as research reagents and diagnostics for the detection of serum antibodies, but also as vaccine candidates and even therapeutics. The S protein has been produced in mammalian cells with a yield of ˜2 mg/ml whereas the much smaller RBD is expressed at higher levels, up to 25 mg/ml (Amanat *et al*., 2020; Zhao et al., 2020). Many groups are now producing variants of this heavily glycosylated protein in plant cell suspension cultures or whole plants by transient expression or in transgenic lines (Makatsa et al., 2020; authors’ unpublished data). Differences in the glycan profiles of S protein variants produced in plants may affect the immunogenicity of different vaccines, contributing to differences in efficacy.

Another key protein used in COVID‐19 research is angiotensin‐converting enzyme 2 (ACE2), which is the major SARS‐CoV‐2 receptor found on pulmonary epithelial cells among others (Hoffmann *et al*., 2020; Wrapp *et al*., 2020). The natural function of ACE2 is the regulation of blood pressure and cardiovascular homeostasis, which is achieved via the proteolytic degradation of the pro‐inflammatory and vasoconstrictive peptide angiotensin II (Ang1‐8) to Ang1‐7 (Anguiano *et al*., 2017). Elevated levels of angiotensin II are pathogenic, so a soluble version of ACE2 (sACE2) is currently in development as a cardiovascular drug and has completed phase I and II trials (Haschke *et al*., 2013; Khan *et al*., 2017). The outbreak of SARS‐CoV‐2 provided impetus for the repurposing of this protein, which was shown to inhibit SARS‐CoV‐2 infection of human cells and organoids *in vitro* and is now undergoing clinical testing (Monteil *et al*., 2020). Like the SARS‐CoV‐2 S protein, human ACE2 is heavily glycosylated, with seven *N‐*linked glycosylation sites and a single *O*‐linked glycosylation site (Shajahan *et al*., 2020). One unique aspect of molecular farming is the distinct glycan profile of plant cell lines compared to mammalian cells (Schoberer and Strasser, 2018), allowing the production of S protein and ACE2 variants as different glycoforms that may differ in terms of stability, immunogenicity and efficacy (Solá and Griebenow, 2010). The absence of ACE2 glycosylation has been shown to impair the uptake of SARS‐CoV‐2 by human cells, with a small impact on the binding affinity (Yang *et al*., 2020). However, a full‐length oral ACE2 expressed in lettuce chloroplasts (thus lacking glycans) accumulates to a 10‐fold higher concentration in the lungs than in plasma, leading to a reduction in right ventricular hypertrophy, total pulmonary resistance index, right ventricle systolic pressure and pulmonary artery remodelling in hypertensive animals (Daniell *et al*., 2020). For COVID‐19 patients, it is important to deliver ACE2 to lung tissues because SARS‐CoV‐2 binds to receptors there and causes significant lung damage. In addition, CTB‐ACE2 with efficient binding to both GM1 and ACE2 can effectively block binding of the S protein and viral entry into human cells, especially via oral epithelial cells that are enriched with both receptors (Xu *et al*., 2020).

Another advantage of molecular farming in the response to COVID‐19 is the speed and scalability of systems based on transient expression in whole plants, which will help to address the massive demand for research reagents, and could ultimately be used to produce vaccines and therapeutics on a sufficient scale for global distribution (Capell *et al*., 2020; Tusé *et al*., 2020). This advantage may also be critical in the future, if variants of SARS‐CoV‐2 evolve to render current vaccine products less effective. Industrial‐scale production will be required for SARS‐CoV‐2 proteins used as vaccine antigens, for sACE2 as a therapeutic and for broadly neutralizing antibodies that may eventually take the place of convalescent serum which is currently being used to treat critically ill patients (Wang *et al*., 2021). Similarly, testing the population for immunity will require billions of serology tests worldwide. Periodic retesting may also become necessary as SARS‐CoV‐2 is already showing signs of becoming an **endemic disease**, and pathogenic coronaviruses may elicit less than 3 years of immunity in some individuals (Long *et al*., 2020; Payne *et al*., 2016). The scalability of production is therefore a key consideration. Currently, recombinant SARS‐CoV‐2 S1 antigen prices fall within the range US$4–25/µg and at least 300 ng of purified antigen is needed for serological tests based on the enzyme‐linked immunosorbent assay (ELISA). One billion tests would therefore cost US$1.2 billion for the antigen alone unless economies of scale can be harnessed. Commercial‐scale production facilities based on transient expression are currently operated by iBio in Bryan, Texas, USA (Holtz *et al*., 2015), Medicago in Quebec, Canada, and Raleigh‐Durham, North Carolina, USA (Lomonossoff and D’Aoust, 2016) and Kentucky Bioprocessing in Owensboro, Kentucky, USA (Pogue *et al*., 2010). All of these facilities are currently being used to produce COVID‐19 reagents and vaccine candidates for clinical testing, including subunit vaccines and VLPs (Capell *et al*., 2020). Medicago has recently published the successful results of phase I trials (Ward *et al*., 2021a) and is currently conducting phase III trials, with 76 million doses already ordered by the Canadian government. Researchers at UC Davis are testing variations of the SARS‐CoV‐2 S protein and ACE2‐Fc fusion proteins in mammalian cells and plant expression systems to compare titre and structures, allowing the immunological analysis of the recombinant glycoproteins combined with molecular dynamics simulations to predict product stability and relative binding affinity (Bernardi *et al*., 2020). Monoclonal antibodies against SARS‐CoV‐2 proteins have also been produced by transient expression in *N. benthamiana* (Rattanapisit *et al*., 2020), and the US company Novici Biotech (Vacaville, CA, USA) has not only produced one such antibody (CR3022) by transient expression but has also donated batches to BEI Resources (Manassas, VA, USA) for distribution at shipping cost only. Transient expression has also been used to produce the antiviral lectin griffithsin (Fuqua *et al*., 2015; Hahn *et al*., 2015), which was shown to act synergistically against SARS‐CoV‐2 (Cai *et al*., 2020). Whereas transient expression allows rapid production, ultimate scalability requires transgenic plants because stable lines can be scaled indefinitely for the long‐term production of proteins in greenhouses or (in some jurisdictions) even in the open field. Other groups, including the authors of this article, are using transgenic systems to produce antiviral lectins in transgenic plants, including rice (Vamvaka *et al*., 2016a,b, 2018) and soybean seeds (O’Keefe et al., 2015).

**Figure 3:**
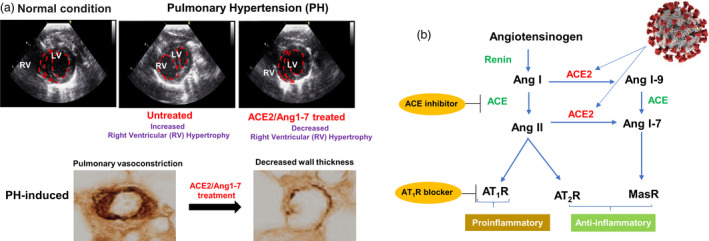
Delivery of ACE2/Ang1‐7 expressed in chloroplasts for the treatment of COVID‐19. (a) Orally delivered ACE2 and its product Ang 1–7 attenuate pulmonary hypertension (PH), reduce RV systolic pressure, RV hypertrophy, fibrosis and pulmonary vessel wall thickness in a rat model, which are the symptoms observed in COVID‐19 patients. (b) SARS‐CoV‐2 binds to the ACE2 receptor in order to enter cells. ACE2 converts Ang I and Ang II to Ang 1–9/Ang 1–7 in the renin–angiotensin system pathway. Oral delivery of plant‐derived ACE2 has the potential to block SARS‐CoV‐2 entry into human cells by competing for the same receptor and also increases the concentration of beneficial Ang 1–7. This figure is modified after Daniel *et al*. (2021). Abbreviations: ACE = angiotensin‐converting enzyme, Ang = angiotensin, AT1R = angiotensin receptor type I, AT2R = angiotensin receptor type II, LV = left ventricle, MasR = Mas receptor, RV = right ventricle

The potential of ACE2/Ang1‐7 to rebalance the severely disrupted renin–angiotensin axis suggests a potential benefit in more severely ill patients with acute respiratory distress syndrome, with or without COVID‐19, where low ACE2 and Ang(1‐7) have been linked to poor prognosis. The NHLBI SMARTT program has already facilitated toxicology, pharmacokinetic and regulatory studies for ACE2/Ang1‐7 expressed in chloroplasts (Figure 3). Briefly, oral dose range finding studies of CTB‐ACE2/CTB‐Ang(1‐7) in healthy male and female Sprague Dawley rats revealed no significant toxicity, and the few mild clinical/laboratory abnormalities were confined to the high‐dose group (Daniell *et al*., 2020). CTB‐ACE2/Ang1‐7 in pharmacokinetic studies was also delivered in a dose‐dependent manner. Clinical studies of CTB‐ACE2/Ang1‐7 are now underway to determine the safety, tolerability and pharmacodynamics of oral ACE2/Ang1‐7 in healthy volunteers (phase 1a) and patients with COVID‐19 (phase 1b). Furthermore, a 2‐week treatment (phase 2) is being evaluated for the ability to improve clinical outcomes in patients admitted to a non‐ICU hospital setting and in patients with COVID‐19 who are triaged to home quarantine, evaluating the median time to release from quarantine using established criteria.

### Seasonal and pandemic influenza

Influenza is a contagious viral infection transmitted by sneezing and coughing. It is caused mainly by influenza viruses A and B, which induce symptoms of fever, myalgia, headache, malaise and the typical signs of upper respiratory infections (coughing, sore throat, congestion). Symptoms typically last 5–10 days but longer in children, the elderly and the immunocompromised. Influenza is recorded in 5–10% of adults and 20–30% of children every year, with both seasonal and epidemic/pandemic patterns. Seasonal influenza occurs in temperate regions, but there is no clear seasonal pattern elsewhere (Moghadami, 2017).

Influenza virus undergoes antigenic drift caused by point mutations but also antigenic shift caused by the recombination of the segmented genome when multiple strains infect the same individual. This leads to rapid changes in the genetic makeup of strains circulating in the human population and new vaccines must therefore be prepared every year (WHO, 2020h). The influenza antigens included in the vaccines are selected based on global surveillance, and decisions are made at least 6 months before the next season to allow time for vaccine manufacturing and approval. Recommendations are proposed by WHO and regional medical regulators for trivalent or quadrivalent formulations including A/H1N1 and A/H3N2 strains, as well as one or two B strains thought most likely to become prevalent in the next season. The viruses are then adapted for propagation in eggs and are produced separately. The long gap between the selection of antigens and the availability of vaccines means that inaccurate predictions can limit the efficacy of the next seasonal vaccine due to the emergence of an unanticipated strain, as occurred between 2010 and 2014 when the effectiveness of the US vaccine dropped from 60% to 19%. Similarly, pandemic strains of influenza emerge occasionally due to zoonotic transmission, as most recently seen in the 2009 swine flu pandemic. The seasonal vaccine offered no immunity against this strain, leading to the emergency manufacture of a new vaccine that included the H1N1/09 antigen.

Plants are promising as a platform for the manufacture of influenza vaccines due to their scalability and the rapid production facilitated by transient expression. Whereas 6 months or more is needed to prepare sufficient quantities of egg‐derived vaccine to meet global demand, the same can be achieved in plants within a few weeks (Shoji *et al*., 2012), as proven in the abovementioned DARPA Blue Angel program that produced 10 million doses in one month (Lomonossoff and D’Aoust, 2016). For this reason, although some influenza antigens have been produced in transgenic plants (Ceballo *et al*., 2017; Firsov *et al*., 2015; Lee *et al*., 2015), most have been transiently expressed in *N. benthamiana* (Hodgins *et al*., 2017; 2019a,b; Landry *et al*., 2014; Shoji *et al*., 2009a,b; Shoji et al., 2013, 2015; Table S1) including all vaccine candidates intended for commercial development. Several products indicated for seasonal and pandemic influenza have already reached late‐stage clinical development, including vaccines against novel influenza strains and quadrivalent vaccines against seasonal variants (Chichester *et al*., 2012; Cummings *et al*., 2014). The candidates have all been based on the hemagglutinin protein (HA), primarily from H1N1 or H5N1 strains of *Influenza A virus*. Various formats have been tested, including monomeric and trimeric HA subunits, but the most popular approach is the presentation of HA on the surface of VLPs. Typical VLPs require a viral coat protein component to form the core of the particle, and influenza vaccines based on tobacco mosaic virus have been developed using this principle by Fraunhofer CMB/iBio (Shoji *et al*., 2011, 2012). These systems can achieve high product yields: for example, vaccines against strains H3N2, H5N1 and H1N1 have been produced at yields of 50–200 mg/kg fresh leaf biomass (Shoji *et al*., 2008, 2011). However, HA can assemble *in planta* and bud from the membrane to form independent enveloped VLPs even in the absence of viral coat protein components (D’Aoust *et al*., 2008) and the particles were found to be immunogenic and protective in mouse challenge studies (D’Aoust *et al*., 2010). This unexpected discovery provided the foundation for the development of multiple vaccine candidates based on carrier‐free VLPs by Medicago, including a proof‐of‐concept vaccine against a newly emerged H7N9 strain (Pillet *et al*., 2015) and a quadrivalent vaccine with HA derived from strains A/California/07/2009 H1N1 (A/H1N1 Cal), A/Victoria/361/11 H3N2 (A/H3N2 Vic), B/Brisbane/60/08 (B/Bris, Victoria lineage) and B/Wisconsin/1/10 (B/Wis, Yamagata lineage), which has reached phase III clinical development (Pillet *et al*., 2016, 2019; Ward *et al*., 2020). The two phase III trials involved a total of 22 930 subjects (Ward *et al*., 2020). The first study of 10 160 adults during the 2017/2018 northern hemisphere influenza season was a placebo‐controlled randomized trial that revealed an absolute vaccine efficacy of 35.1%, which was lower than the primary endpoint of the study (70% efficacy) but better than the performance of the standard quadrivalent vaccine in the same year, particularly when comparing the response against the H3N2 strain. The second study of 12,794 adults aged 65 or more during the 2018/2019 northern hemisphere influenza season was a direct comparison of the plant‐derived and egg‐derived vaccines, showing the former had an 8.8% higher efficacy despite the lower antibody response. A third phase III trial was recently completed successfully using VLPs based on strains A/California/07/2009 H1N1, A/Hong Kong/4801/2014 H3N2, B/Brisbane/60/08 and B/Phuket/3073/2013 (recommended for the 2016–2017 Northern Hemisphere season) in 1200 healthy adults aged 18–49 years to test lot‐to‐lot consistency and to confirm safety and immunogenicity (Ward *et al*., 2021b). Overall, the influenza VLPs have been shown to interact with macrophages similarly to the native virus, which may explain their superior efficacy compared to killed vaccines adapted in eggs (Makarkov *et al*., 2017, 2019). At the cellular level, the VLPs were shown to present trimeric HA that interacted directly with dendritic cells in humans and mice, promoting the release of pro‐inflammatory cytokines and the activation of antigen‐specific T‐cell responses (Won *et al*., 2018).

The strong immunogenicity of HA‐based VLPs has led to a wealth of clinical data concerning the specific immune response to these vaccine candidates and has, as a by‐product, helped to address one of the early contentious aspects of molecular farming – the impact of plant glycans. The clinical testing of the H5N1 influenza vaccine is pertinent because the phase I/II trials specifically measured the human antibody response to *N*‐linked glycans present on the VLP surface. In the phase I trial, seven of 48 subjects (14.6%) already produced such antibodies (Landry *et al*., 2010) and in the extended phase I/II trial of the same product, the frequency of seropositive subjects before vaccination was 19.2% (Ward *et al*., 2014). A total of 280 of 349 subjects received either one or two intramuscular doses of vaccine, including 40 subjects with pre‐existing plant allergies. Subjects were monitored for 6 months after vaccination, and 34% developed transient IgG responses to glycans. Some subjects developed IgE responses but not to the glycoepitope containing xylose and fucose, which is specific to plants, and there was no evidence of allergy or hypersensitivity (Landry *et al*., 2010; Ward *et al*., 2014). Similar results were reported in phase I/II trials of the quadrivalent influenza vaccine. Only two of 30 subjects tested positive for IgE against the glycoepitope during the study, and one subject with pre‐existing IgE actually lost the IgE response following vaccination, most probably reflecting background exposure to environmental plant carbohydrates. Approximately 24 000 people have now received one or two doses of the plant‐derived VLP vaccine without the induction or worsening of clinical allergies, the development of pathological IgE responses or the long‐term elicitation of glycan‐specific IgG. The development of influenza vaccines produced by transient expression is therefore one of the key niches of molecular farming that show unambiguous advantages over all other current manufacturing platforms, and this is reflected by the keen interest and investment in the platform by the US and Canadian governments.

### Ebola haemorrhagic fever

Ebola haemorrhagic fever in humans is caused by four of the six known species of *Ebolavirus*, with *Zaire ebolavirus* causing the most severe symptoms and the greatest number of fatalities. The first known outbreak was in 1976 in Sudan, although the cause was not known until several months later when a second outbreak occurred in Zaire (now the Democratic Republic of the Congo). The virus spreads through contact with body fluids and is thought to transfer from fruit bats to primates (including humans) and some domestic animals. There have been six major outbreaks in the last decade, some of which are current, and the fatality rate for infected individuals is ˜50% on average, with a maximum of ˜90% (WHO, 2020d). Work on vaccine development was intermittent until the recent outbreaks brought the disease to global attention, particularly the threat of wider spreading due to international travel, but it has been challenging to complete vaccine trials due to the disease tailing off before programmes can be mounted, leaving a shortage of recruits (Chappell and Watterson, 2017). Three vaccines have progressed to efficacy trials after successful phase I safety evaluation, two of which were based on adenovirus vectors expressing the *Zaire ebolavirus* glycoprotein 1 (ChAd3‐ZEBOV and Ad26‐EBOV/MVA‐EBOV) and one based on recombinant vesicular stomatitis virus (rVSV‐EBOV). The adenovirus vaccines are efficacious in non‐human primates, although Ad26‐EBOV/MVA‐EBOV requires a modified vaccinia Ankara booster dose. The rVSV‐EBOV vaccine was also shown to be effective in humans in a delayed deployment efficacy trial conducted in Guinea at the tail end of the 2014/2015 outbreak (Chappell and Watterson, 2017) and was administered to more than 300 000 enrollees in the subsequent 2018 outbreak in the Democratic Republic of the Congo, resulting in 80% of vaccine recipients resisting the disease and the remainder showing only mild symptoms (Maxmen, 2020).

Given the success of the rVSV‐EBOV vaccine and the restriction of vesicular stomatitis virus to mammalian cells, molecular farming has not been used to independently develop any Ebola vaccine candidates. A chimeric protein containing two epitopes from the *Zaire ebolavirus* glycoprotein 1 fused to the nontoxic B component of the enterotoxigenic *Escherichia coli* (ETEC) heat‐labile toxin (LTB) was expressed in tobacco (14.7 µg/kg fresh leaf biomass) and was immunogenic in BALB/c mice, producing IgA and IgG responses following oral immunization (Ríos‐Huerta et al., 2017), but this was not developed any further perhaps because the yields were too low for an economically viable process. However, plants have been used to produce monoclonal antibodies against the virus which can help with the treatment of patients who have already contracted the disease (Olinger *et al*., 2012; Zeitlin *et al*., 2011). Single monoclonal antibodies and cocktails of two or three different types have been produced in *N*. *benthamiana* and transplastomic lettuce leaves, achieving the neutralization of Ebola virus in pre‐clinical tests (Fulton *et al*., 2015; Lai *et al*., 2012; Lin *et al*., 2018; Phoolcharoen *et al*., 2011). The light and heavy chains of monoclonal antibody 6D8 were produced with yields of up to 0.5 g/kg by transient expression (Phoolcharoen *et al*., 2011). A cocktail of three monoclonal antibodies known as ZMapp (c13C6, c2G4 and c4G7) was also transiently expressed in tobacco leaves (0.5 g/kg) and, for each antibody, the full‐size tetrameric IgG complex containing two heavy and two light chains retained its virus‐binding activity (Qiu *et al*., 2014). It is important to note that the three antibodies were expressed separately and the purified products were subsequently mixed – the simultaneous expression of multiple antibodies in the same plant is likely to result in hybrid immunoglobulin assembly. In another study, three anti‐Ebola virus mouse/human chimeric monoclonal antibodies (c13C6, h‐13F6 and c6D8) produced in tobacco completely protected two infected animals in pre‐clinical tests (Olinger *et al*., 2012). ZMapp was subsequently approved under the animal efficacy rule, which allows temporary use in humans based on animal data if clinical studies would be impossible or unethical. This was deemed to be the case in the 2014–2016 Ebola outbreak in West Africa, in which seven patients were treated with ˜9 g of the cocktail and five survived (Qiu *et al*., 2014). Conventional clinical development continued in parallel, but as in previous vaccine studies it was not possible to collect sufficient data to achieve statistical significance because the number of patients declined as the epidemic wound down (PREVAIL II Writing Group et al., 2016). In 2020, the FDA approved Inmazeb for the treatment of *Zaire ebolavirus* infection in adults and children, a combination of three monoclonal antibodies (atoltivimab, maftivimab and odesivimab) produced in CHO cells (Markham, 2021). The lectin CV‐N has also shown animal efficacy against Ebola virus (Barrientos *et al*., 2003).

### Zika fever

The mosquito‐transmitted Zika virus was discovered in East Africa in 1947, and sporadic individual infections in humans were reported from 1954, but the first epidemic did not occur until 2007, when 185 cases were recorded in the Yap Islands of Micronesia (Gubler *et al*., 2017). A series of outbreaks in 2013–2014 affected ˜9000 people across Oceania, followed by a major epidemic in 2015–2016 in Brazil, spreading to the rest of the Americas and affecting up to 35 000 people, garnering international attention and a WHO classification as a Public Health Emergency of International Concern. Although direct fatalities from the disease are unknown, mother‐to‐child transmission during pregnancy can cause microcephaly and other brain malformations in the foetus (Rasmussen *et al*., 2016). Several countries that have experienced Zika outbreaks have also reported increases in the prevalence of Guillain‐Barré syndrome, which causes muscle weakness and in some cases breathing difficulties and cardiovascular complications. The properties of Zika fever, Ebola fever and non‐seasonal influenza as epidemic diseases are compared in Table 3.

**Table 3:** Differences between H5N1, Ebola and Zika (modified from Park and Wi, 2016). R_0_ is the basic reproduction number, the expected number of cases directly generated by one case in a population where all individuals are susceptible to the disease

	H5N1	Ebola	Zika
Period	1997‐present	1976‐present	1947‐present
Natural host	Birds	African fruit bats, gorillas, chimpanzees, and other mammals	Monkeys
Intermediate host	Unknown	Unknown	Female mosquitoes, primarily *Aedes aegypti* and *Aedes albopictus*
Transmission method	From infected birds to human; rare human‐to‐human spread	Close contact with the blood, secretions, organs or other bodily fluids of infected animals and humans	Primarily through the bites of infected female mosquitoes, but also via body fluids
R_0_	0.05–2.68	1.5–1.9	3.8
Incubation period	2–9 days	3–21 days	3–14 days
Case fatality rate (average)	60%	50%	8.3%
Most common symptoms	An influenza‐like illness of fever, cough, and shortness of breath; severe respiratory disorders leading to death	Fever, fatigue, muscle pain, headache, sore throat; internal and external bleeding	Fever, chills, generalized myalgia, cough, shortness of breath, nausea, vomiting and diarrhoea; also associated with Guillain‐Barré syndrome.

A number of Zika virus vaccine candidates are currently under development, many based on previously approved platforms and designs against dengue and other infectious diseases (Tripp and Ross, 2016). For example, the Zika envelope protein (ZE3) is a safe and efficacious vaccine candidate, producing VLPs that elicit robust anti‐ZE3 antibody titres and neutralize the virus efficiently without an adjuvant (Diamos *et al*., 2020b). The transient expression of ZE3 in *N. benthamiana* achieved yields of up to 160 mg/kg fresh leaf biomass, suggesting this could be an effective response strategy to further outbreaks (Yang *et al*., 2018). As discussed for Ebola, transient expression has also been used to produce single monoclonal antibodies and cocktails of two or three different types against Zika virus. The yield was up to 1.5 g/kg fresh leaf biomass, and the purified antibodies retained their ability to recognize the Zika virus envelope protein and neutralize the virus (Diamos *et al*., 2020a).

## Molecular farming as a strategy to address established pandemic diseases

### HIV/AIDS

HIV is a retrovirus that is transmitted through sexual intercourse or contact with infected blood. The virus targets cells of the immune system (CD4^+^ T cells, macrophages and dendritic cells) and therefore leads to an acquired immunodeficiency syndrome (AIDS) that renders the patient susceptible to opportunistic infections, which are generally the direct cause of death (Bowen *et al*., 2016; Buchacz *et al*., 2016;). Although there is no vaccine or cure, HIV loads can be controlled by antiretroviral drugs, particularly highly active antiretroviral therapy (HAART) involving multiple drugs with different targets. Accordingly, the population living with HIV is steadily increasing and was nearly 38 million in 2018, with eastern and southern Africa the worst affected regions (UNAIDS, 2020).

HIV is a global disease, but its effects are disproportionately felt in poorer regions without access to drugs or preventatives, so molecular farming in transgenic plants is a particularly suitable approach given the potential for large‐scale inexpensive production and the use of crops that can be grown locally in developing countries. Several HIV proteins have been expressed in plants as potential vaccine candidates, including the gp140 envelope protein in *N*. *benthamiana* at 21.5 mg/kg fresh leaf biomass, which was well tolerated and immunogenic in rabbits (Margolin *et al*., 2019, 2020). HIV coat protein genes have also been transiently expressed in *N*. *glutinosa* and *N. benthamiana*, with yields of 814.2 and 462.6 µg/kg fresh leaf biomass, respectively (Ataie Kachoie *et al*., 2018). The p24 capsid protein (and a fusion of p24 with the negative regulatory protein Nef) was expressed in transplastomic tobacco and tomato plants to levels of up to 40% of total protein and proved to be immunogenic following subcutaneous and oral administration in experimental animals (Gonzalez‐Rabade *et al*., 2011; McCabe *et al*., 2008; Zhou *et al*., 2008). HIV‐specific antibodies have been expressed in transgenic rice (Vamvaka *et al*., 2016c), tobacco (Niemer *et al*., 2014) and maize (Sabalza *et al*., 2012) and have shown promising neutralizing activity *in vitro*, suggesting they could be used as microbicidal formulations (Ramessar *et al*., 2008b). An HIV polyepitope has also been expressed in lettuce (Govea‐Alonso *et al*., 2013). Antiviral lectins such as cyanovirin‐N and griffithsin transiently expressed in *N. benthamiana* (Habibi *et al*., 2018) or stably expressed in soybean (O’Keefe et al., 2015), rice (Vamvaka *et al*., 2016a,b) and transplastomic tobacco (Elghabi *et al*., 2011; Hoelscher *et al*., 2018) have also proven effective. The benefits of the soybean system are expanded in Box 2.

Vamvaka *et al*. (2018) expressed the HIV‐neutralizing monoclonal antibody 2G12 and two lectins (cyanovirin‐N and griffithsin) in the same transgenic rice line, providing an inexpensive and scalable strategy for the provision of microbicidal cocktails. The high mutation rate of HIV means that single components are ineffective due to the evolution of escape mutants, but combinations of three or more components targeting different viral epitopes can help to prevent viral spreading. The rice system is advantageous because typical molecular farming products must be purified to homogeneity, and this would abolish the advantage of the triple transgenic lines. However, the generally regarded as safe (GRAS) status of rice and other cereals means that microbicides could be prepared from crude extracts (essentially ground seeds) allowing the development of effective microbicide cocktails that can be stored and transported as dry seed without a cold chain and then prepared as a microbicide in a local facility (Vamvaka *et al*., 2018). As a model for this approach, transgenic rice expressing recombinant proteins is already grown in the field in locations where there are no rice crops or compatible wild species, such as the Ventria facilities in the USA and US Virgin Islands (APHIS, 2018), and other companies grow seed‐based crops in containment for the same purposes (Fischer and Buyel, 2020). Transgenic tobacco plants expressing 2G12 were developed during the EU‐funded Pharma‐Planta project, which resulted in a successful phase I clinical trial (Ma *et al*., 2015). In this case, the production platform and regulatory aspects were adjusted during the study as the regulations concerning GMP requirements changed during the project, resulting in the accreditation of a GMP facility and the successful production of clinical‐grade plant‐derived 2G12 for intravaginal testing in healthy female subjects (Ma *et al*., 2015; Sack *et al*., 2015). As stated above, griffithsin has also been produced by transient expression (Fuqua *et al*., 2015; Hahn *et al*., 2015) and the economic benefits of this approach have been discussed (Alam *et al*., 2018).

Box 2 **The production of lectins in soybean seeds**

Cyanovirin‐N is a lectin isolated from the cyanobacterium *Nostoc ellipsosporum*, and it has been shown to inactivate SIV and HIV by establishing multiple weak interactions with the surface envelope glycoprotein (gp120) thus blocking membrane fusion (Boyd *et al*., 1997; Tsai *et al*., 2003;). In the context of molecular farming, it is important to note that bacterial systems are uneconomical for the production of this protein because high yields of functional protein are difficult to achieve given the dependence on multiple disulfide bonds (Lofti *et al*., 2018). Transgenic plants offer a scalable alternative (O’Keefe et al., 2015). Soybean seeds are particularly attractive due to their endogenous protein content of ˜40% compared to 8–10% for cereals (Stoger *et al*., 2005; Vianna *et al*., 2011b). In soybean seeds, the protein storage vacuoles (PSVs) are involved in the accumulation of ˜70% of all seed proteins (Shimada *et al*., 2018; Tan *et al*., 2019;) making them ideal for the accumulation of recombinant proteins (Cunha *et al*., 2011a,b, 2013, 2014a,b, 2019, 2020; Jolliffe *et al*., 2005; Rech *et al*., 2014; Tan *et al*., 2019; Vianna *et al*., 2011a,b; Wakasa and Takaiwa, 2013). Multiple recombinant proteins have therefore been expressed in soybean PSVs, including proinsulin (Cunha *et al*., 2010), human growth hormone (Cunha *et al*., 2011a), coagulation factor IX (Cunha *et al*., 2011b, 2014b), cyanovirin‐N (O’Keefe et al., 2015), antibodies (Vianna *et al*., 2011a,b) and tumour antigens (Rech *et al*., 2014). The proteins remained stable and active after 7 years of seed storage at room temperature (Cunha *et al*., 2011a,b; O’Keefe et al., 2015; Rech *et al*., 2014; Vianna *et al*., 2011a). Cyanovirin‐N was expressed in soybean using the β‐conglycinin promoter to restrict expression to the seeds and a signal peptide to target the PSVs, resulting in the detection of the protein specifically in the seeds 4 weeks after pollination, with peak expression after 8 weeks (O’Keefe et al., 2015). The final concentration of pure cyanovirin‐N in the seeds was 3% of total soluble protein (350 g/kg dry seed biomass), and no recombinant protein was detected in the seed oil.

### Human papillomavirus

HPV is a sexually transmitted virus that is harmless in ˜90% of cases but in a minority of infected persons causes warts that increase the risk of cancer of the anus, vulva, vagina, penis and oropharynx (IARC, 2020). More than 200 HPV genotypes have been grouped according to the viral genome structure and tropism to human epithelial tissues (Garbuglia, 2014). Twelve high‐risk types (HPV16, 18, 31, 33, 35, 39, 45, 51, 52, 56, 58 and 59) are classified as carcinogenic to humans, leading to 570 000 cases of cervical cancer and 311 000 deaths in 2018 (IARC, 2020). HPV is combatted by a combination of preventative vaccination and screening (Arbyn *et al*., 2020). Several vaccines have been developed, mostly targeting oncoproteins E6 and E7 (Chabeda *et al*., 2018; Hung *et al*., 2008; Ma *et al*., 2010; Moniz *et al*., 2003) by aiming to deliver the E6 and E7 antigens in to APCs in order to activate CD8^+^ cytotoxic T cells or CD4^+^ helper T cells (Yang *et al*., 2016). Bivalent and quadrivalent HPV vaccines containing HPV16 and HPV18 antigens protect naïve individuals with high efficacy and are therefore administered preferentially to school‐age girls (Arbyn *et al*., 2020).

The current VLP‐based HPV vaccines – Cervarix (GlaxoSmithKline) and Gardasil (Merck) – are highly effective but expensive to produce because they are manufactured in insect and yeast cells, respectively (Wang and Roden, 2013). They also require a cold chain and must be delivered by intramuscular injection (Ma *et al*., 2013). Several plant‐based systems have therefore been explored, including transient expression in *N. benthamiana*, and transgenic tobacco, potato, Arabidopsis and tomato plants. The primary targets have been the L1 and L2 capsid proteins (Buck *et al*., 2013) and the E6 and E7 oncoproteins (De La Rosa *et al*., 2009; Franconi *et al*., 2002; Massa *et al*., 2007; Morgenfeld *et al*., 2009). The L1 and L2 proteins were produced as VLPs in transgenic tobacco and potato (Biemelt *et al*., 2003; Varsani *et al*., 2003). Codon optimization of the HPV sequence and the introduction of a tobacco mosaic virus translational enhancer increased the L1 yields to 20 mg/kg fresh potato tuber, and all the VLPs were immunogenic in mice (Biemelt *et al*., 2003) and rabbits (Varsani *et al*., 2003) in the presence of adjuvants (Biemelt *et al*., 2003; Varsani *et al*., 2003; Warzecha *et al*., 2003). Transient expression in *N*. *benthamiana* (Liu *et al*., 2005) and expression in transgenic Arabidopsis (Kohl *et al*., 2007) and transplastomic tomato (De La Rosa *et al*., 2009) did not improve efficacy or yields. However, a codon‐optimized HPV‐16 L1 protein was produced by transient expression in *N. benthamiana* with a yield of 550 mg/kg fresh leaf biomass (Regnard *et al*., 2010), and in tobacco plastids with yields of 887 mg/kg (Maclean *et al*., 2007) and 3 g/kg fresh leaf biomass, the latter equivalent to 24% total soluble protein (Fernández‐San Millán *et al*., 2008). In other transplastomic plants, the yield was ˜1.5% of the total soluble protein (Lenzi *et al*., 2008; Waheed *et al*., 2011). The L2 protein has only been expressed as an L1‐L2 fusion and the yields were no higher than reported above for L1 (Čeřovská *et al.*, 2008, 2012; Chabeda *et al*., 2019; Lamprecht *et al*., 2016; Matić *et al*., 2012). The E7 protein was produced by transient expression in *N. benthamiana* using potato virus X vectors, with yields of 3–4 g/kg fresh leaf biomass (Franconi *et al*., 2002). E7 was also expressed as a fusion to the PVX coat protein, increasing the stability and immunogenicity of the product compared to native E7 by allowing the assembly of multivalent structures (Morgenfeld *et al*., 2009). E7 was also expressed as a fusion to the bacterial enzyme lichenase, improving its stability and facilitating purification, leading to yields of ˜100 mg/kg fresh leaf biomass (Buyel *et al*., 2012).

### Hepatitis

Viral hepatitis is an inflammation of the liver caused by one of five related viruses named hepatitis viruses A–E (Jefferies *et al*., 2018). Transmission depends on the type of virus, but it is mainly through sexual contact (HBV), contaminated blood (HCV) or contaminated food and water caused by inadequate sanitation (HAV and HEV). Almost one third of the global population has been infected by either HBV or HCV (WHO, 2012), and 1.34 million people died as a result in 2015 (WHO, 2017b). The highest prevalence of hepatitis is in sub‐Saharan Africa for HAV and HBV (Jefferies *et al*., 2018), the Eastern Mediterranean Region and Europe for HCV (WHO, 2017b), whereas HEV is more common in Asia and the rest of Africa (Razavi, 2020). HDV only infects individuals already infected with HBV because it requires HBV for replication (Razavi, 2020). Effective vaccines are available against HAV and HBV, but not HCV or HDV (Duncan *et al*., 2020; Ogholikhan and Schwarz, 2016), and the current HEV vaccine is only licenced for use in China (Zhang *et al*., 2015) but has not been approved by the FDA (Ogholikhan and Schwarz, 2016). Lectins have also shown activity against HCV, including griffithsin administered by subcutaneous injection (Meuleman et al., 2011; Takebe *et al*., 2013).

The HBV surface antigen (HBsAg) was the first vaccine produced in tobacco and was antigenically and physically similar to the HBsAg spherical particles derived from human serum and recombinant yeast (Mason *et al*., 1992). Several further subunit vaccines have since been expressed in plants, including the core antigens of HCV (HCcAg) and HBV (HBcAg) as well as antibodies against HBsAg (Hernández‐Velázquez *et al*., 2015; Huang *et al*., 2006; Mohammadzaeh et al., 2015). Tobacco has been used as the expression host in most studies (López *et al*., 2008; Nemchinov *et al*., 2000; Rukavtsova *et al*., 2007; Zhou *et al*., 2006) but also fruits and vegetables such as tomato (Lou *et al*., 2007; Ma *et al*., 2003b), banana (Elkholy *et al*., 2009), lettuce (Clarke *et al*., 2017; Dobrica *et al*., 2018; Kapusta *et al*., 1999), carrot (Imani *et al*., 2002) and potato (Thanavala *et al*., 2005), and cereals such as rice (Qian *et al*., 2008). Two studies have led directly to human clinical trials involving oral vaccines in edible vegetable tissues. Three human volunteers were fed with transgenic lettuce leaves expressing HBsAg (first 200 g, then another 150 g within 2 months). The yield of HBsAg in the lettuce leaves was 1–5 µg/kg fresh tissue. Two weeks after the second dose, HBsAg‐specific antibody levels > 10 IU/L were detected in sera from two of three volunteers, which is the accepted minimum protective level against HBV (Kapusta *et al*., 1999). In the second study, 33 human volunteers previously immunized with the licenced HBV vaccine (with current antibody titres ≤115 IU/L) were fed with transgenic potato tubers containing HBsAg at a concentration of 8.5 ± 2.1 mg/kg fresh biomass. The volunteers ingested 100–110 g of raw tuber in two doses (days 0 and 28) or three doses (days 0, 14 and 28), causing the antibody titres to increase up to 56‐fold after three doses in 10 of 16 volunteers and up to 33‐fold after two doses in 9 of 17 volunteers (Thanavala *et al*., 2005). Several pre‐clinical studies have demonstrated immune responses in mice against plant‐derived antigens of HAV (Chung *et al*., 2011, 2014), HBV (Dobrica *et al*., 2018; Huang *et al*., 2006), HCV (Nemchinov *et al*., 2000) and HEV (Zhou *et al*., 2006).

Interestingly, as well as its role as a vaccine candidate, HBcAg is also regarded as a model protein for the testing of molecular farming strategies to increase yields. It has therefore been produced by transient expression (Mechtcheriakova *et al*., 2006) and has also been directed to accumulate in the plastids (Zhou *et al*., 2006), vacuole (Hayden *et al*., 2012) and endoplasmic reticulum (Chung *et al*., 2014; Mohammadzadeh *et al*., 2015) of transgenic plants. In the latter case, low proteolytic activity and the presence of chaperones to promote folding resulted in higher levels of accumulation (Tremblay *et al*., 2010). However, the highest reported accumulation of HBcAg was in transgenic tobacco when the construct was driven by the cauliflower mosaic virus 35S promoter (CaMV35S) and *nos* terminator, without a signal peptide, resulting in a yield of 110–250 g/kg fresh biomass (Pyrski *et al*., 2017). The HBcAg protein from this experiment induced an immune response in mice, with a 2–5 times more effective response at a dose of 2 × 10 µg rather than 2 × 1 µg (Pyrski *et al*., 2017). As discussed elsewhere in this article, the high stability of the HBV core antigen has been exploited by using it as a fusion partner for less stable antigens, allowing the development of chimeric VLPs displaying proteins from other viruses (Peyret *et al*., 2015).

## The future of molecular farming for epidemic and pandemic diseases

Scientific advances drive the potential contributions of the international plant science community in the fight against infectious diseases, but this alone is insufficient to guarantee success. As with product development, technology platform development and implementation are highly reliant on acceptability, accessibility, engagement and capacity, particularly if the involvement of low‐to‐middle‐income countries (LMICs) is envisaged (Ma *et al*., 2013). In the case of epidemic or pandemic diseases, the involvement of LMIC partners would be a key component of any sustainable long‐term action. One example is the long‐term collaboration between Bharathiar University (Coimbatore, India) and St. George’s University (London, UK), which has been supported by the government‐funded UK‐India Education and Research Initiative since 2007. Through a series of relatively small awards, these two universities established a collaboration targeting two important infectious diseases (Chikungunya and dengue), promoting exchange visits by project leaders and graduate students and four 2‐day workshops on molecular farming with sponsorships for graduate scientists from across India to attend. From an Indian national perspective, through the UKIERI‐funded workshops, more than 300 graduate scientists were introduced to molecular farming and were able to engage regularly with national experts from research organizations, industry and government regulators, to plan the development path for plant molecular farming in India. The key outputs thus far have been knowledge dissemination and human capacity building across India, which must now be followed up with further investment into plant‐based manufacturing (Murad *et al*., 2020).

On a broader scale, molecular farming must overcome the barriers of industry inertia and regulatory nonalignment that currently prevent its widespread commercial uptake and coordinated international large‐scale deployment for vaccine and biologics manufacturing. Molecular farming is a disruptive technology that subverts industry norms, in which biopharmaceutical production is an entirely cleanroom‐based process with all biological materials, equipment and reagents, and thus the entire upstream and downstream process, meeting the requirements of GMP. In contrast, the upstream portion of molecular farming often utilizes whole plants grown in indoor facilities under controlled conditions using quality‐controlled raw materials and reagents or, depending on the product, under good agricultural and collection practices (GACP) rather than GMP. The downstream process, beginning after the plant homogenization/extraction step and extending through the production of final drug product, would need to meet GMP requirements. The regulation of plant‐made pharmaceuticals in the United States and Europe, as well as considerations during public health emergencies, has been recently reviewed by Tusé *et al*., (2020) illustrating the need to streamline and unify regulatory procedures globally. These elements must be addressed before molecular farming can become a mainstream technology platform and the world can benefit from its capacity for rapid, large‐scale and lower cost production (Nandi *et al*., 2016), which appears to offer the only realistic approach for global coordinated high‐volume vaccine and biologics manufacturing when faced with an **emerging** disease such as COVID‐19. The emergency regulations adopted by the FDA and EMA to facilitate COVID‐19 drug development could also help to facilitate this transition by providing a framework in which effective solutions are prioritized over the rigorous enforcement of regulations designed to govern non‐emergency drug development (Tusé *et al*., 2020). If these barriers can be overcome, molecular farming offers hope to billions of people around the world suffering from endemic, epidemic and pandemic diseases.

## Conflicts of interest

The authors declare no conflicts of interest.

## Author contributions

PC and TC conceived the topic. All authors contributed sections to this article based on their expertise and experience, provided comments and recommendations on the combined draft and approved the final version.

## Supporting information


**Table S1** Classification of epidemic and pandemic diseases based on their epidemiology
