# Supplementary material for: Contributions of the international plant science community to the fight against human infectious diseases – part 1: epidemic and pandemic diseases
Source: Plant Biotechnol J. 2021 Jul 19;19(10):1901–20. doi: 10.1111/pbi.13657 (PMC8486245; doi:10.1111/pbi.13657)
Supplement: Supplementary file 1 — Table S1 Classification of epidemic and pandemic diseases based on their epidemiology [file PBI-19-1901-s001.docx]

**Supplementary files**

Supplementary Table 1. Classification of epidemic and pandemic diseases based on their epidemiology.

| **Disease** | **Prevalence** | **Current treatments** | **Vaccine available?** |
| --- | --- | --- | --- |
| Severe Acute Respiratory Syndrome (SARS) | In November 2002, a SARS outbreak originated in China and spread worldwide. A total of 8,098 cases were reported with a mortality of 9.6% (774 deaths). Most of the cases were reported in China (5,327), Hong Kong, SAR, China (1,755), and Taiwan (346). No human cases have been detected over the past 15 years, after the SARS epidemic ended in July 2003 [1]. | Oxygen (if needed) and isolation [2] | No |
| Middle East Respiratory Syndrome (MERS) | From April, 2012 to January, 2020, a total of 2,519 confirmed human cases of MERS were detected worldwide, with a mortality of 34.3% (866 deaths). The majority of the cases (2,121, with 788 deaths) were in Saudi Arabia [1,3,4]. | Isolation and treat symptoms (anti-inflammatory drugs) [2] | No |
| Severe Acute Respiratory Syndrome-Coronavirus 2 (SARS2) (COVID-19) | More than 180 million cases of SARS-CoV-2 have been detected worldwide, with more than 3.9 million deaths (2.1% mortality) by July 2021. More than half of the confirmed cases are in the America, followed by Europe, and South-East Asia [5,6]. | Isolation, treat symptoms (anti-inflammatory drugs), mechanical ventilation (if needed) [2] | Yes [7–9] |
| Asian Avian Influenza A (H5, H7, and H9 virus flu) | Infections with H7N9 were first reported in March, 2013 in China. China has experienced a total of 6 epidemics of H7N9 human infections. In the fifth and largest epidemic a total of 766 human infections were reported. Since October, 2017 only 3 confirmed cases were reported in China. The cumulative number of infections since 2016 is 1,565, with a mortality rate of approximately 39% [10]. One infection with H7N2 was reported in New York City with a person that had contact with a sick cat [11].  From 2003 to December, 2020, 862 cases of H5N1 were confirmed in 15 countries, with a mortality of 53% (455 deaths) [12]. On January, 2014 a human infection with H5N1 was reported in Canada, in a traveller from China [13]. From December, 2014 to March, 2015, 164 people were exposed to domestic H5 virus in USA, 63%, 34%, and 2% were associated with H5N2, H5N8, and H5N1 virus, respectively. Up to date, Egypt, Indonesia and Vietnam reported the highest number of H5N1 cases in humans [14].  Sporadic infections with H9N2 were reported in China, Bangladesh, and Egypt, commonly in children [13]. | Antiviral treatment [15] | Yes [16] |
| Ebola | There have been 28 outbreaks with Ebola virus in Africa since 1976 [17]. The outbreak with the highest number of cases (28,652) was from March, 2014 to June, 2016 in Guinea, Sierra Leone, and Liberia. The mortality rate was of 39.5% (11,325 deaths) [18]. The last outbreak in Democratic Republic of Congo and Uganda started on August, 2018 and finished on June, 2020. There were a total of 3,470 cases with a mortality rate of 66% (2,287 deaths) [19]. | Intravenous body salts, oxygen, and treat symptoms (anti-inflammatory drugs) [20] | Yes [21–25] |
| Zika | Until 2007, only 14 human cases infected with Zika have been reported worldwide. The first large outbreaks were on the island of Yap (Federated States of Micronesia) in 2007 and in the Pacific Islands from 2013 to 2014. In 2016 there was an outbreak in the Americas, in Brazil a total of 200,000 cases were reported with 8,000 babies born with malformations due to the Zika virus infection. In the USA a total of 45,680 cases of Zika infection were detected in 2016. From then, the incidence peak declined substantially [26–28]. [28] | Treat symptoms and prevent dehydration [29] | No |
| Hepatitis | There are 1.4 million estimated cases of hepatitis A every year [30]. Hepatitis A caused approximately 11,000 deaths in 2015 [31]. In the USA, since 2016 there are hepatitis A outbreaks due to people who inject drugs. A total of 12,474 cases were reported in 2018, with an estimation of 24,900 cases due to people that don’t get diagnosed [32,33]. [33]  A total of 257 million people is estimated to live worldwide with hepatitis B [32]. In 2015, worldwide prevalence of hepatitis B was 3.5%. The highest number of cases was in the Western Pacific Region (approx. 115 million cases), followed by the African Region (approx. 60 million cases) [31].  It was estimated that in 2015 there were 1.75 million new infections with hepatitis C. Injection drugs are one of the reasons of the new infections [31]. In 2015, worldwide prevalence of hepatitis C was 1%, 71 million people were living with chronic hepatitis C. The highest number of people infected was in the Eastern Mediterranean Region, the Western Pacific Region, and the European Region (all of them with approx. 15 million of cases) [31].  It was estimated that a total of 5% of the hepatitis B infected patients are co-infected with hepatitis D [31]. Mongolia, the Republic of Moldova, and Western and Middle Africa countries have a high prevalence of hepatitis D [34,35].  There is an estimation of 20 million infections of hepatitis E worldwide every year [31]. In 2015, 44,000 deaths were estimated to be due to hepatitis E. Hepatitis E is distributed worldwide but most commonly in East and South Asia [36]. | HAV: no specific treatment [37]  HBV: injected PEGylated interferon-alpha [37]  HCV: chemotherapy (injected PEGylated interferon and ribarin ingestion) or liver transplantation [37] | Yes [38] |
| Human Immunodeficiency Virus (HIV) | A total of 38 million people were infected with HIV in 2019, with a mortality of 1.8% (690,000 deaths). Africa is the region with most number of infections (25.7 million infected people), followed by America and South-East Asia (both with 3.7 million infected people). At the end of 2019, 39 million people were living with HIV [39]. | Antiretroviral therapy (combination of HIV medicines) to slow viral progression in the body [40,41] | Yes [42,43] |
| Human Papillomavirus (HPV) | HPV is very common, at some moment every sexually active human gets it. A total of 528,000 estimated new cases occurred in 2012, with 266,000 estimated deaths. The 85% of the cases occurred in developing countries [44]. In USA, from 2013 to 2014 the prevalence of high-risk HPV for adults from 18 to 59 years old was 45.2 % [45]. | No specific treatment, only treatment of health problems caused by HPV [46] | Yes [47] |
| Seasonal influenza | In 2020, about 450,000 cases of influenza virus have been reported worldwide until May, 64% of the cases were Influenza A infections (about 290,000) and 35% were Influenza B (about 160,000 infections). Most of the cases were reported in North America (about 270,000 cases), followed by South West Europe (about 60,000 cases) and Eastern Asia (about 35,000 cases) [48] | Antiviral treatment [49] | Yes [50,51] |

**References**

1. Mernish ZA, Perlman S, Van Kerkhove MD, Zumla A (2020) Middle East respiratory syndrome. Lancet 395:1063–1077.

2. Medscape (2020) Coronaviruses and Acute Respiratory Syndromes (COVID-19, MERS, and SARS) - Infectious Diseases - MSD Manual Professional Edition. <https://www.msdmanuals.com/professional/infectious-diseases/respiratory-viruses/coronaviruses-and-acute-respiratory-syndromes-covid-19,-mers,-and-sars>.

3. Petrosillo N, Viceconte G, Ergonul O, Ippolito G, Petersen E (2020) COVID-19, SARS and MERS: are they closely related? Clinal Microbiol Infect 26:729–734.

4. WHO (2020) MERS situation update, January 2020 | MERS-CoV | Epidemic and pandemic diseases. <http://www.emro.who.int/pandemic-epidemic-diseases/mers-cov/mers-situation-update-january-2020.html>.

5. WHO (2021) COVID-19 situation reports. <https://www.who.int/emergencies/diseases/novel-coronavirus-2019/situation-reports>.

6. WHO (2021) Coronavirus Disease (COVID-19) Dashboard. <https://covid19.who.int/>.

7. FDA (2020) Vaccines and Related Biological Products Advisory Committee Meeting. <https://www.fda.gov/downloads/AdvisoryCommittees/CommitteesMeetingMaterials/BloodVaccinesandOtherBiologics/VaccinesandRelatedBiologicalProductsAdvisoryCommittee/UCM583779.pdf>.

8. Jackson LA, Anderson EJ, Rouphael NG, Roberts PC, Makhene M, Coler RN, McCullough MP, Chappell JD, Denison MR, Stevens LJ, Pruijssers AJ, McDermott A, Flach B, Doria-Rose NA, Corbett KS, Morabito KM, O’Dell S, Schmidt SD, Swanson PA, Padilla M, Mascola JR, Neuzil KM, Bennett H, Peters E, Makowski M, Albert J, Cross K, Buchanan W, Pikaart-Tautge R, Ledgerwood JE, Graham BS, Beigel JH (2020) An mRNA Vaccine against SARS-CoV-2 — Preliminary Report N Engl J Med 383:1920–1931.

9. Pfizer, Biontech (2020) Emergency Use Authorization (EUA) of the Pfizer-Biontech COVID-19 Vaccine to Prevent Coronavirus Disease 2019 (COVID-19) in Individuals 16 Years of Age and Older.

10. CDC (2018) Asian Lineage Avian Influenza A(H7N9) Virus | Avian Influenza (Flu). <https://www.cdc.gov/flu/avianflu/h7n9-virus.htm>.

11. CDC (2016) Avian Influenza A (H7N2) in Cats in Animal Shelters in NY; One Human Infection. <https://www.cdc.gov/flu/spotlights/avian-influenza-cats.htm>.

12. WHO (2020) Cumulative number of confirmed human cases of avian influenza A(H5N1) reported to WHO (Table 9 December 2020). <https://www.who.int/influenza/human_animal_interface/2020_DEC_tableH5N1.pdf?ua=1>.

13. CDC (2015) Avian Influenza Current Situation Summary | Avian Influenza (Flu). <https://www.cdc.gov/flu/avianflu/avian-flu-summary.htm>.

14. CDC (2015) Update: Domestic H5 Outbreak in Birds. <https://www.cdc.gov/flu/news/update-h5-outbreak-birds.htm>.

15. CDC (2017) Prevention and Treatment of Avian Influenza A Viruses in People | Avian Influenza (Flu). <https://www.cdc.gov/flu/avianflu/prevention.htm>.

16. FDA (2018) H5N1 Influenza Virus Vaccine, manufactured by Sanofi Pasteur, Inc. Questions and Answers. <https://www.fda.gov/vaccines-blood-biologics/vaccines/h5n1-influenza-virus-vaccine-manufactured-sanofi-pasteur-inc-questions-and-answers>.

17. CDC (2021) Ebola Virus Disease Distribution Map: Cases of Ebola Virus Disease in Africa Since 1976. <https://www.cdc.gov/vhf/ebola/history/distribution-map.html>.

18. CDC (2021) 2014-2016 Ebola Outbreak Distribution in West Africa. <https://www.cdc.gov/vhf/ebola/history/2014-2016-outbreak/distribution-map.html?CDC_AA_refVal=https%3A%2F%2Fwww.cdc.gov%2Fvhf%2Febola%2Foutbreaks%2F2014-west-africa%2Fdistribution-map.html>.

19. CDC (2020) 2018 Eastern Democratic Republic of the Congo Ebola Outbreak Map | Democratic Republic of Congo | Outbreaks | Ebola (Ebola Virus Disease). <https://www.cdc.gov/vhf/ebola/outbreaks/drc/east-drc-map.html>.

20. CDC (2021) Treatment | Ebola (Ebola Virus Disease). <https://www.cdc.gov/vhf/ebola/treatment/index.html>.

21. Henao-Restrepo AM, Longini IM, Egger M, Dean NE, Edmunds WJ, Camacho A, Carroll MW, Doumbia M, Draguez B, Duraffour S, Enwere G, Grais R, Gunther S, Hossman S, Kondé MK, Kone S, Kuisma E, Levine MM, Mandal S, Norheim G, Riveros X, Soumah A, Trelle S (2015) Efficacy and effectiveness of an rVSV-vectored vaccine expressing Ebola surface glycoprotein: interim results from the Guinea ring vaccination cluster-randomised trial. Lancet 386:857–866.

22. Henao-Restrepo AM, Camacho A, Longini IM, Watson CH, Edmunds WJ, Egger M, Carroll MW, Dean NE, Diatta I, Doumbia M, Draguez B, Duraffour S, Enwere G, Grais R, Gunther S, Gsell PS, Hossmann S, Watle SV, Kondé MK, Kéita S, Kone S, Kuisma E, Levine MM (2017) Efficacy and effectiveness of an rVSV-vectored vaccine in preventing Ebola virus disease: final results from the Guinea ring vaccination, open-label, cluster-randomised trial (Ebola Ça Suffit!). Lancet 389:505–518.

23. WHO (2019) Preliminary results on the efficacy of rVSV-ZEBOV-GP Ebola vaccine using the ring vaccination strategy in the control of an Ebola outbreak in the Democratic Republic of the Congo: an example of integration of research into epidemic response.

24. CDC (2020) Prevention and Vaccine | Ebola (Ebola Virus Disease). <https://www.cdc.gov/vhf/ebola/prevention/index.html>.

25. Sebastian S, Flaxman A, Cha KM, Ulaszewska M, Gilbride C, Sharpe H, Wright E, Spencer AJ, Dowall S, Hewson R, Gilbert S, Lambe T (2020) A Multi-Filovirus Vaccine Candidate: Co-Expression of Ebola, Sudan, and Marburg Antigens in a Single Vector. Vaccines 8:241.

26. BMJ Best Practice (2020) Zika virus infection - Epidemiology. <https://bestpractice.bmj.com/topics/en-gb/1302/epidemiology>.

27. CDC (2019) Statistics and Maps | Zika virus. <https://www.cdc.gov/zika/reporting/index.html>.

28. Da Cunha AJLA, De Magalhães-Barbosa MC, Lima-Setta F, De Andrade Medronho R, Prata-Barbosa A (2016) Microcephaly case fatality rate associated with Zika virus infection in Brazil. Pediatr Infect Dis J 36:528–530.

29. CDC (2019) Treatment | Zika virus. <https://www.cdc.gov/zika/symptoms/treatment.html>.

30. WHO (2015) Hepatitis A. <http://www.who.int/immunization/diseases/hepatitisA/en/>.

31. WHO (2017) Global hepatitis report, 2017.

32. CDC (2020) Global Viral Hepatitis: Millions of People are Affected <https://www.cdc.gov/hepatitis/global/index.htm>.

33. CDC (2019) Hepatitis A | Epidemiology of Vaccine Preventable Diseases. <https://www.cdc.gov/vaccines/pubs/pinkbook/hepa.html>.

34. WHO (2020) Hepatitis D. <https://www.who.int/news-room/fact-sheets/detail/hepatitis-d>.

35. Medscape (2020) Roy PK, Mujibur R, Kanth R, Lacey SR: Hepatitis D: Background, Etiology, Epidemiology. <https://emedicine.medscape.com/article/178038-overview>.

36. WHO (2020) Hepatitis E. <https://www.who.int/news-room/fact-sheets/detail/hepatitis-e>.

37. UCSF Health (2020) Viral Hepatitis Treatment <https://www.ucsfhealth.org/conditions/viral-hepatitis/treatment>.

38. Ogholikhan S, Schwarz KB (2016) Hepatitis vaccines. Vaccines 4.

39. WHO (2020) HIV/AIDS. <https://www.who.int/data/gho/data/themes/hiv-aids>.

40. AIDSinfo (2020) HIV Treatment: The Basics | Understanding HIV/AIDS. <https://aidsinfo.nih.gov/understanding-hiv-aids/fact-sheets/21/51/hiv-treatment--the-basics>.

41. CDC (2020) Treatment | Living with HIV | HIV Basics | HIV/AIDS. <https://www.cdc.gov/hiv/basics/livingwithhiv/treatment.html>.

42. Pitisuttithum P, Rerks-Ngarm S, Bussaratid V, Dhitavat J, Maekanantawat W, Pungpak S, Suntharasamai P, Vanijanonta S, Nitayapan S, Kaewkungwal J, Benenson M, Morgan P, O'Connell RJ, Berenberg J, Gurunathan S, Francis DP, Paris R, Chiu J, Stablein D, Michael NL, Excler JL, Robb ML, Kim JH (2011) Safety and reactogenicity of canarypox ALVAC-HIV (vCP1521) and HIV-1 gp120 AIDSVAX B/E vaccination in an efficacy trial in Thailand. PLoS One 6.

43. Gray GE, Laher F, Lazarus E, Ensoli B, Corey L (2016) Approaches to Preventative and Therapeutic HIV vaccines. Curr Opin Virol 17:104–109.

44. WHO (2020) Human papillomavirus (HPV). <https://www.who.int/immunization/diseases/hpv/en/>.

45. CDC (2021) HPV Statistics. <https://www.cdc.gov/std/hpv/stats.htm>.

46. CDC (2016) HPV Treatment. <https://www.cdc.gov/std/hpv/treatment.htm>.

47. NIH (2019) Human Papillomavirus (HPV) Vaccines - National Cancer Institute. <https://www.cancer.gov/about-cancer/causes-prevention/risk/infectious-agents/hpv-vaccine-fact-sheet>.

48. CDC (2020) 2019-2020 U.S. Flu Season: Preliminary Burden Estimates. <https://www.cdc.gov/flu/about/burden/preliminary-in-season-estimates.htm>.

49. CDC (2020) Flu Treatment. <https://www.cdc.gov/flu/treatment/index.html>.

50. CDC (2020) Summary of Recommendations. <https://www.cdc.gov/flu/professionals/acip/summary/summary-recommendations.htm>.

51. CDC (2021) Seasonal Influenza Vaccination Resources for Health Professionals. <https://www.cdc.gov/flu/professionals/vaccination/index.htm>.
